# The Effect of Cadmium on COX-1 and COX-2 Gene, Protein Expression, and Enzymatic Activity in THP-1 Macrophages

**DOI:** 10.1007/s12011-015-0234-6

**Published:** 2015-02-03

**Authors:** Tomasz Olszowski, Izabela Gutowska, Irena Baranowska-Bosiacka, Katarzyna Piotrowska, Jan Korbecki, Mateusz Kurzawski, Dariusz Chlubek

**Affiliations:** 1 Department of Hygiene and Epidemiology, Pomeranian Medical University, Powstańców Wlkp. 72 Av, 70-111 Szczecin, Poland; 2 Department of Biochemistry and Human Nutrition, Pomeranian Medical University, Broniewskiego 24 Str, 71-460 Szczecin, Poland; 3 Department of Biochemistry and Medical Chemistry, Pomeranian Medical University, Powstańców Wlkp. 72 Av, 70-111 Szczecin, Poland; 4 Department of Physiology, Pomeranian Medical University, Powstańców Wlkp. 72 Av, 70-111 Szczecin, Poland; 5 Department of Experimental and Clinical Pharmacology, Pomeranian Medical University, Powstańców Wlkp. 72 Av, 70-111 Szczecin, Poland

**Keywords:** Cadmium, Cyclooxygenase-1, Cyclooxygenase-2, Prostaglandin E_2_, Thromboxane B_2_, THP-1 macrophages

## Abstract

The aim of this study was to examine the effects of cadmium in concentrations relevant to those detected in human serum on cyclooxygenase-1 (COX-1) and cyclooxygenase-2 (COX-2) expression at mRNA, protein, and enzyme activity levels in THP-1 macrophages. Macrophages were incubated with various cadmium chloride (CdCl_2_) solutions for 48 h at final concentrations of 5 nM, 20 nM, 200 nM, and 2 μM CdCl_2_. The mRNA expression and protein levels of COXs were analyzed with RT-PCR and Western blotting, respectively. Prostaglandin E_2_ (PGE_2_) and stable metabolite of thromboxane B_2_ (TXB_2_) concentrations in culture media were determined using ELISA method. Our study demonstrates that cadmium at the highest tested concentrations modulates COX-1 and COX-2 at mRNA level in THP-1 macrophages; however, the lower tested cadmium concentrations appear to inhibit COX-1 protein expression. PGE_2_ and TXB_2_ production is not altered by all tested Cd concentrations; however, the significant stimulation of PGE_2_ and TXB_2_ production is observed when macrophages are exposed to both cadmium and COX-2 selective inhibitor, NS-398. The stimulatory effect of cadmium on COXs at mRNA level is not reflected at protein and enzymatic activity levels, suggesting the existence of some posttranscriptional, translational, and posttranslational events that result in silencing of those genes’ expression.

## Introduction

Cyclooxygenase-1 (COX-1) and cyclooxygenase-2 (COX-2) are the bifunctional enzymes catalyzing the conversion of arachidonic acid (AA) to prostaglandin H_2_ (PGH_2_) in two sequential reactions, the first being the generation of prostaglandin G_2_ (PGG_2_) (cyclooxygenase reaction) followed by the reduction of PGG_2_ to PGH_2_ (peroxidase reaction). The generated PGH_2_ is the precursor of biologically active prostanoids such as prostaglandin D_2_ (PGD_2_), prostaglandin E_2_ (PGE_2_), prostaglandin I_2_ (PGI_2_), prostaglandin F_2α_ (PGF_2α_) and thromboxane A_2_ (TXA_2_) [1, 2]. COX-1 and COX-2 enzymes share 60 % identity in their amino acid sequences [3]. They exist as homodimers; each subunit consists of three domains, the epidermal growth factor domain, the membrane binding domain, and the catalytic domain containing the cyclooxygenase and peroxidase active sites [3].

COX-1, constitutively expressed in almost all cell types, but also inducible in some systems [4], was previously considered to be involved in physiological processes and playing no role in inflammation [4]. However, according to newer concept, COX-1 is also involved in inflammatory process [2, 5]; for example, COX-1 not only is responsible for the initial prostanoid response to inflammatory stimuli [6] but also contributes to the resolution of inflammation [4]. The human COX-1 gene (*Ptgs1*) expression is developmentally controlled and can be upregulated by tumor-promoting phorbol esters or growth factors [7]. The regulatory elements of this gene include SP1 binding site and activator protein-1 (AP-1) site [4, 7]; however, the transcriptional control of COX-1 gene expression was not well studied [7]. The products of COX-1 enzyme are thromboxane A_2_ (being metabolized to its stable metabolite, TXB_2_) and (PGE_2_ [6].

COX-2 is an enzyme highly inducible by pro-inflammatory cytokines, tumor promoters, mitogens, and growth factors in a variety of cell types, including monocytes [8], which results in increased prostaglandin release [5]. According to newer concept, COX-2 is the major contributor to prostanoid synthesis as inflammation progresses [6]. COX-2 gene (*Ptgs2*) contains several potential transcriptional regulatory elements in the 5′-flanking region: peroxisome proliferator response element (PPRE), two nuclear factor kappa B (NF-κB) sites, one specificity protein 1 (Sp1) site, two cyclic AMP response elements (CRE), one nuclear factor for interleukin-6 expression (NF-IL6) motif, two AP-1 sites, E-box, and TATA box [4, 7, 9]. Transcriptional regulation of COX-2 gene is very complex; it can involve numerous signaling pathways, and the mechanism varies depending on the specific stimulus and the cell type [7]. The main product of COX-2 enzyme is PGE_2_ [6].

Cadmium is a toxic and carcinogenic heavy metal that poses nowadays a serious threat to human health because it is ubiquitously distributed in the environment and the food, tobacco smoke, and ambient air constitute the most significant sources of cadmium exposure for the general population [10, 11]. Cadmium was found to be immunomodulator; it may modify cell-mediated and humoral immune response, which may be associated with the occurrence of allergic, inflammatory diseases, and cancers [12, 13]. The target cells for heavy metals action such as cadmium are lymphocytes and macrophages, which participate in humoral immune response. As a result of toxic action of cadmium, the pro-inflammatory, pro-coagulatory, and chemotactic factors are released, activating macrophages to produce cytokines and to development of the further stages of immune reaction [12]. Cadmium was found to cause upregulation of some mediators and markers of inflammation [11]. A number of studies investigated the effect of cadmium on COX-2 mRNA [8, 14–19], protein expression [14, 16, 18, 20–25], and enzymatic activity [16–18, 24, 26, 27]. Most of them demonstrated a stimulatory effect of this metal on COX-2 in different experimental models [8, 16–19, 21–27]. However, a few reports suggested cadmium to exert either inhibitory action [14, 15] or no effect [20] on COX-2. The impact of cadmium on COX-1 was rather poorly analyzed in literature: only a few studies dealt with this issue, suggesting either no [16, 28] or stimulatory effect [17].

THP-1 cells model has some advantages over human macrophages isolated from blood of cadmium-exposed people. Their homogenous genetic background minimizes the degree of variability in the cell phenotype [29]. Such cell model eliminates the influence of other environmental factors that may interfere with the examined mechanisms of cadmium action. Therefore, THP-1 cells experimental system represents a convenient model for the studies of molecular mechanisms of cadmium action on macrophages in relation to inflammatory processes [29].

The aim of this study was to examine the effects of cadmium in low concentrations (relevant to levels detected in human serum) on activity and expression of COX-1 and COX-2.

## Materials and Methods

### Materials

The materials used include anti-mouse IgG FITC conjugated (Sigma-Aldrich, Poland), antibiotics (penicillin and streptomycin) (Sigma-Aldrich, Poland), Bakerbond columns (Witko Group, Poland), cadmium chloride (Sigma-Aldrich, Poland), cDNA Reverse Transcription Kit (Life Technologies, USA), COX-1 and COX-2 mouse monoclonal antibody (Santa Cruz, Germany), FBS (ALAB, Poland), goat anti-mouse IgG-HRP (Santa Cruz, Germany), Micro BCA Protein Assay kit (Thermo Scientific, USA), monoclonal anti-β-actin antibody(1:200; clone AC-74, Sigma Aldrich, Poland), nitrocellulose membrane (Thermo Scientific, Pierce Biotechnology, USA), NS-398 (Sigma-Aldrich, Poland), PBS (Biomed-Lublin, Poland), phorbol 12-myristate13-acetate (PMA) (Sigma-Aldrich, Poland), Precision Plus Protein Kaleidoscope Standards (Bio-Rad, Poland), Prostaglandin E_2_ EIA Kit (Cayman, USA), RNAqueous Mini Kit (Life Technologies, USA), RPMI medium (Biomed-Lublin, Poland), Super Signal West Pico Chemiluminescent Substrate (ALAB, Poland), Taqman Gene Expression Assays (Applied Biosystems, USA), THP-1 cells (American Type Culture Collection ATCC, Rockville, USA), and Thromboxane B_2_ EIA Kit (Cayman, USA).

### Cell Culture and Treatment

The experiments were conducted on macrophages derived from a human monocytic cell line THP-1. The differentiation of THP-1 cells into macrophages was achieved by administration of 100 nM PMA and further incubation for 24 h. The adherent macrophages were washed three times with PBS and then incubated with cadmium chloride (CdCl_2_) solutions for 48 h at 37 °C in 5 % CO_2_. The following concentrations of CdCl_2_ were used in this study: 5 nM, 20 nM, 200 nM, and 2 μM. They were selected based on the cadmium levels found in human serum [13]. In half of the culture dishes, the cadmium-exposed macrophages were additionally incubated with COX-2 selective inhibitor, NS-398 (50 μM). After 48 h, the cells were harvested by scraping and the pellets were obtained by centrifugation (800 × *g*, 10 min). Afterwards, the cool PBS was added to the pellets and the samples were stored at −80 °C until the following further analyses: the measurement of protein concentration using Micro BCA Protein Assay Kit (Thermo Scientific, Rockford, USA). The remaining supernatants were placed in new tubes and stored at −80 °C until further analyses, that is the extraction and measurement of PGE_2_ and TXB_2_ by ELISA method.

### Cyclooxygenase-1 and Cyclooxygenase-2 Gene Expression Analysis by qRT-PCR

The quantitative analysis of the expression of *Ptgs1* and *Ptgs2* genes was performed in a two-step reverse transcription PCR. Total RNA was extracted from cells using RNAqueous Mini Kit (Life Technologies, USA). The quantity and quality of isolated RNA were determined using the Nanodrop ND-1000 spectrophotometer (NanoDrop Technologies, USA). cDNA was prepared from 400 ng of total cellular RNA in 20 μl of reaction volume, using High capacity cDNA Reverse Transcription Kit (Life Technologies, USA) with random primers, according to manufacturer’s instructions. Quantitative real-time PCR was performed in 7500 Fast Real-Time PCR System (Applied Biosystems, USA), using pre-validated Taqman Gene Expression Assays (Applied Biosystems, USA) and a FAM-labeled probe for analyzed genes and a VIC-labeled probe for endogenous control gene: *GAPDH*, TaqMan GE Master Mix (Life Technologies, USA) and 1.5 μl of cDNA for each reaction mix of 15 μl. Every sample was analyzed simultaneously in two technical replicates; the mean *C*_T_ values were used for further investigation. The relative quantification method was applied in calculations, using 7500 Fast Real-Time PCR System Software (Applied Biosystems, USA). The thresholds were set manually to compare data between runs and *C*_T_ values were extracted. All *C*_T_ values were normalized to the mean for endogenous controls (*GAPDH*) for each sample. Analysis of these relative changes in gene expression between samples was performed using the 2^−ΔΔC^_T_ method.

### The Measurements of Cyclooxygenase-1 and Cyclooxygenase-2 Expression by Western Blotting

Scraping of cells was followed by lysis using lysing buffer (protease inhibitor, ethylenediaminetetraacetic acid 5 mM; Sigma Aldrich, Poland) and cell lysates were collected in −80 °C. Separation of equal amounts of protein (20 μg) was performed in 10 % sodium dodecylsulfate (SDS)/polyacrylamide gel electrophoresis followed by transfer to a nitrocellulose membrane (Thermo Scientific, Pierce Biotechnology, USA) at 157 mA for 1.5 h at room temperature. After blocking the membrane with 5 % (COX-1) or 3 % (COX-2) non-fat milk in Tris-buffered saline (Sigma Aldrich, Poland) containing 0.1 % Tween 20 (Sigma Aldrich, Poland) for 1 h at room temperature, it was incubated with primary antibodies direct against COX-1 and COX-2 (1:200; Santa Cruz Biotechnology, USA) or with a monoclonal anti-β-actin (1:200; clone AC-74, Sigma Aldrich, Poland) and next with secondary antibodies (goat anti-mouse IgG HRP, 1:2,000; Santa Cruz Biotechnology, USA). Signals were visualized by chemiluminescence (Thermo Scientific, Pierce Biotechnology, USA).

### The Measurements of Prostaglandin E_2_ and Thromboxane B_2_ Concentrations

PGE_2_ and TXB_2_ were extracted from culture supernatants using Bakerbond columns (Witko Group, Poland). The measurements of PGE_2_ and TXB_2_ levels were conducted using appropriate immunoenzymatic sets (Prostaglandin E_2_ EIA Kit, Cayman, USA; Thromboxane B_2_ EIA Kit, Cayman, USA) according to manufacturers’ instruction.

### Imaging of Cyclooxygenase-1 and Cyclooxygenase-2 Expression

Expression of COX-1 and COX-2 proteins was examined with confocal microscopy. THP-1 macrophages were grown on cover glasses in standard in vitro culture conditions. Further, cells were washed with PBS and fixed with 4 % buffered formalin for 15 min in room temperature. After the fixation and washing with PBS, cells were permeabilized with 0.5 % solution of Triton X-100 in PBS. After washing with fresh portion of PBS, cells were incubated with primary antibodies: mouse anti-COX-1 and mouse anti-COX-2 (Santa Cruz Biotechnology) in 1:50 dilution, in 4 °C, overnight and then washed and incubated with secondary antibody: anti-mouse IgG FITC conjugated, dilution 1:60 (Sigma-Aldrich) in antibody diluent (Dako), 30 min in room temperature and after washing with PBS further with Hoechst 33258, 30 min, room temperature. The cells were examined under a confocal microscope (FV1000 confocal with IX81 inverted microscope, Olympus, Germany); three channel acquisition and sequential scanning were used for best resolution of signal from Hoechst 33258 and FITC fluorescence. Additionally, fluorescent images were merged with transition light images.

### Statistical Analysis

The statistical analysis of obtained results was conducted using Statistica 10 software (Statsoft, Poland). The results were expressed as arithmetical mean ± standard deviation (SD). The distribution of variables was evaluated using Shapiro-Wilk *W* test. The nonparametric tests were used for further analyses because distribution in most cases deviated from normal distribution. The results were subjected to Wilcoxon matched-pair test. The level of significance was set at *p* < 0.05.

## Results

### Cadmium at Highest Tested Concentrations Increases Cyclooxygenase-1 mRNA Expression, While at Low Tested Concentrations Decreases Protein Expression in THP-1 Macrophages

**Fig. 1:**
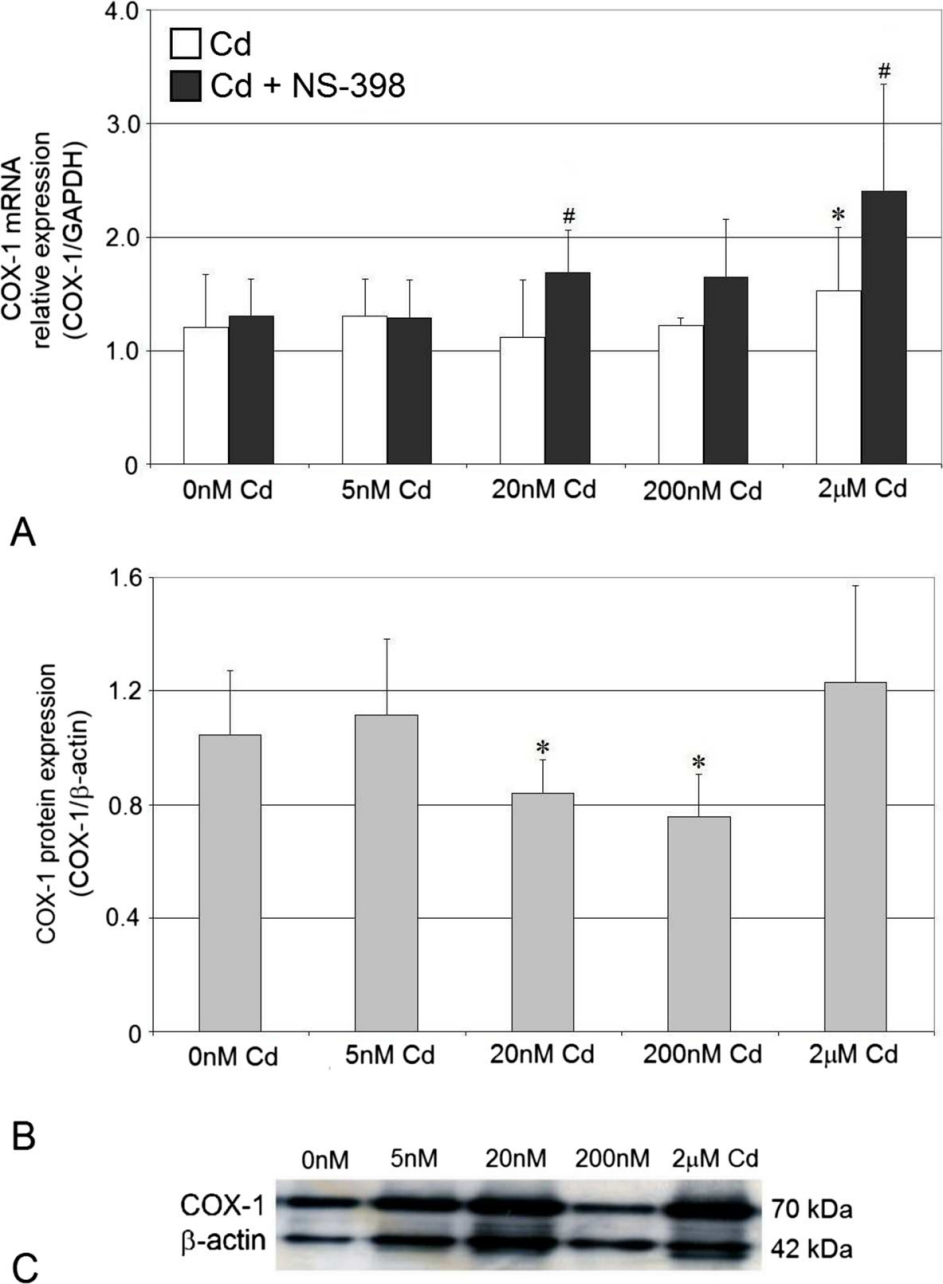
The effect of cadmium on COX-1 mRNA and protein expression in macrophages cultured with various cadmium solutions. **a** COX-1 mRNA expression following cadmium exposure without or with addition of COX-2 selective inhibitor, NS-398; **b** COX-1 protein expression (densitometric analysis of protein normalized to β-actin; **c** representative Western blot following cadmium exposure. Monocytes/macrophages were cultured with cadmium solutions for 48 h. After incubation, cells were harvested by scraping and mRNA was measured by using real-time PCR method (*n* = 4) and protein expression by using Western blotting method (*n* = 3). *Asterisk*, statistically significant as compared with 0 nM Cd—cells incubated in RPMI medium with 10 % FBS and with DMSO addition (Wilcoxon test). *Number sign*, statistically significant as compared with the experiment 0 nM Cd with NS-398 (Wilcoxon test)

In macrophages cultured with CdCl_2_, the mRNA expression of COX-1 significantly increased (35 %) (*p* = 0.043) for 2 μM cadmium solution (*p* = 0.043) (Fig. 1). Addition of NS-398 (COX-2 selective inhibitor) to cultures caused significant upregulation of COX-1 mRNA for 20 nM (*p* = 0.043) and 2 μM (*p* = 0.043) cadmium solution (29 and 84 % increase, respectively).

**Fig. 2:**
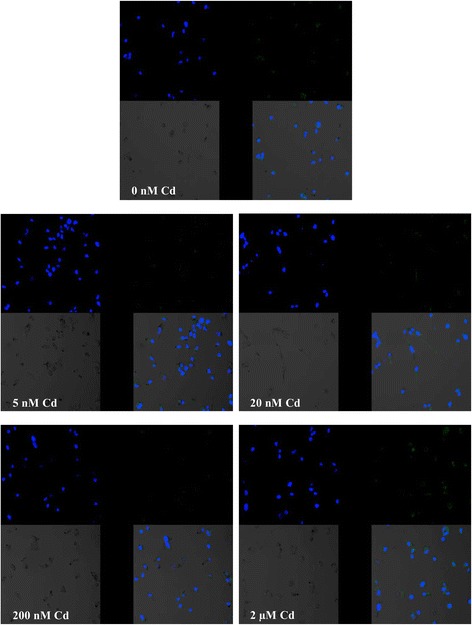
Imaging of COX-1 enzyme by fluorescence microscopy in macrophages cultured with cadmium. Monocytes/macrophages were cultured with Cd solutions for 48 h. The immunohistochemistry was performed using specific primary antibody, mouse anti-COX-1 (the overnight incubation at 4 °C), and secondary antibodies conjugated with flouorochrome–anti-mouse IgG FITC (incubation for 45 min at room temperature). The nuclei of cells were DAPI stained. Image analysis was performed with a fluorescent microscope using filters 38 HE GFP for green fluorescence and 49 DAPI for blue fluorescence

The estimation of the effects of cadmium on COX-1 protein expression was performed using Western blot and immunocytochemistry. The results obtained using these two methods are consistent. COX-1 protein expression decreased markedly following exposure to 20 nM (*p* = 0.012) and 200 nM (*p* = 0.012) cadmium solution (19.5 and 27 % decrease, respectively) (Fig. 1b and c). The images taken by fluorescence microscopy confirmed the influence of cadmium solution on the decrease in COX-1 protein expression (Fig. 2).

### Cadmium at Highest Tested Concentrations Increases Cyclooxygenase-2 mRNA Expression, While It Exerts No Effect on Protein Levels in THP-1 Macrophages

**Fig. 3:**
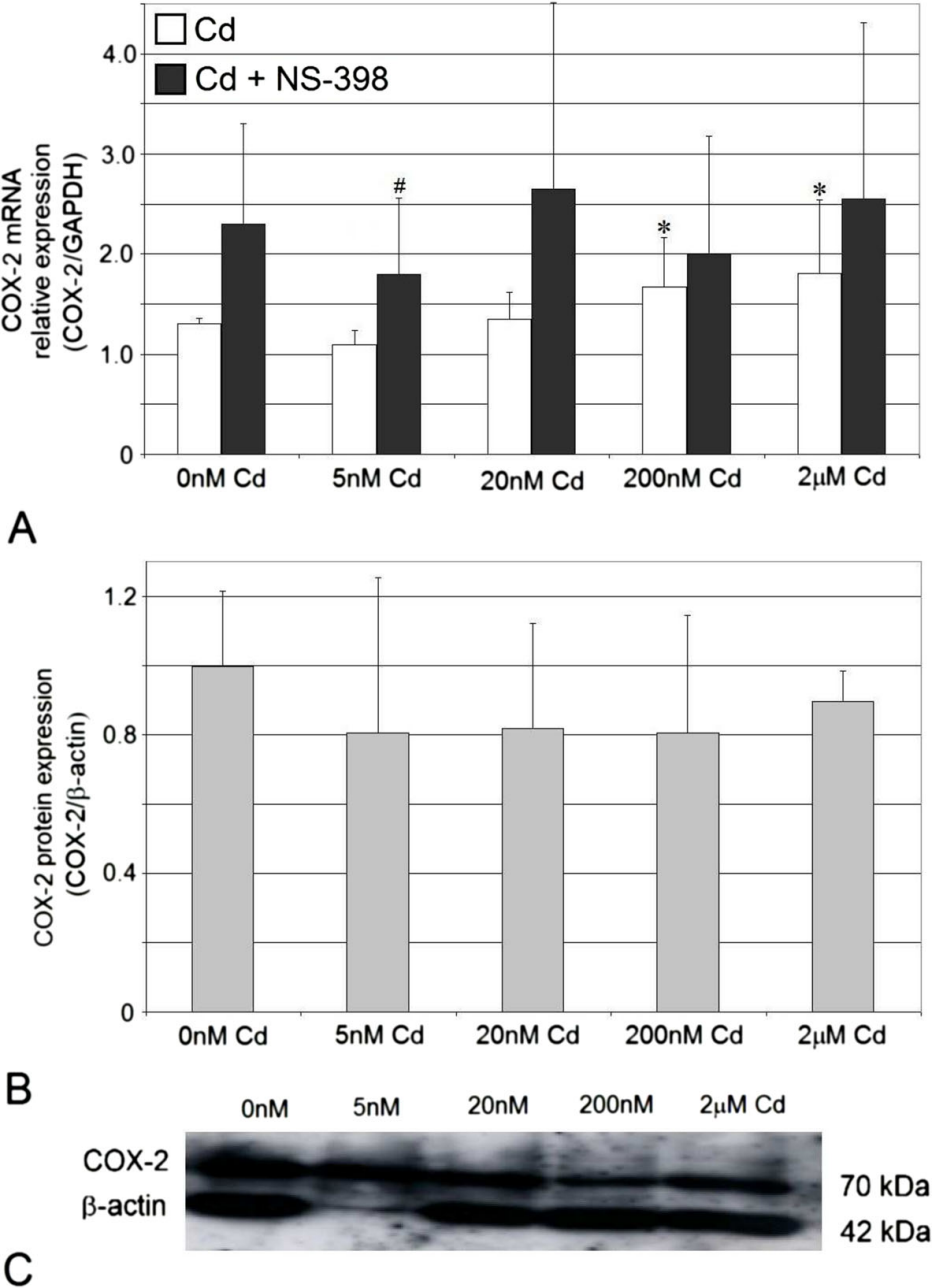
The effect of cadmium on COX-2 mRNA and protein expression in macrophages cultured with various cadmium solutions. **a** COX-2 mRNA expression following cadmium exposure without or with addition of COX-2 selective inhibitor, NS-398; **b** COX-2 protein expression (densitometric analysis of protein normalized to β-actin; **c** representative Western blot following cadmium exposure. Monocytes/macrophages were cultured with cadmium solutions for 48 h. After incubation, cells were harvested by scraping and mRNA was measured by using real-time PCR method (*n* = 4) and protein expression by using Western blotting method (*n* = 3). *Asterisk*, statistically significant as compared with 0 nM Cd—cells incubated in RPMI medium with 10 % FBS and with DMSO addition (Wilcoxon test). *Number sign*, statistically significant as compared with the experiment 0 nM Cd with NS-398 (Wilcoxon test)

COX-2 mRNA expression increased in a cadmium concentration-dependent manner, with significant upregulation for 200 nM (*p* = 0.043) and 2 μM (*p* = 0.027) cadmium solution (18.5 and 40 % increase, respectively) (Fig. 3a). Addition of COX-2 selective inhibitor, NS-398 to cultures did not modulate significantly COX-2 mRNA expression at most cadmium concentrations tested; however, the interaction of cadmium at very low concentration (5 nM; *p* = 0.046) and NS-398 resulted in significant downregulation of COX-2 mRNA expression (*p* = 0.046) (22 % decrease).

**Fig. 4:**
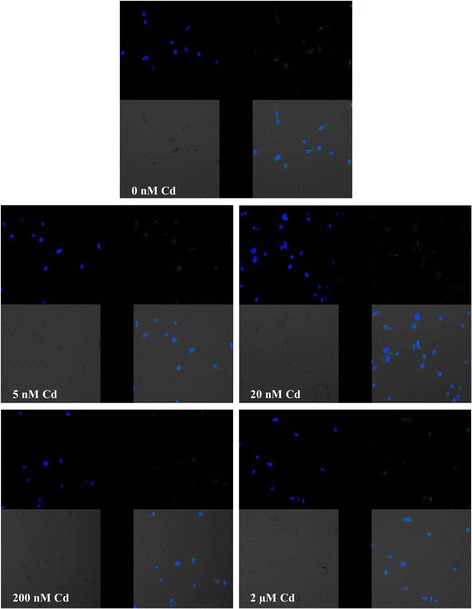
Imaging of COX-2 enzyme by fluorescence microscopy in macrophages cultured with cadmium. Monocytes/macrophages were cultured with Cd solutions for 48 h. The immunohistochemistry was performed using specific primary antibody, mouse anti-COX-2 (the overnight incubation at 4 °C), and secondary antibodies conjugated with flouorochrome–anti-mouse IgG FITC (incubation for 45 min at room temperature). The nuclei of cells were DAPI stained. Image analysis was performed with a fluorescent microscope using filters 38 HE GFP for green fluorescence and 49 DAPI for blue fluorescence

The estimation of the effects of cadmium on COX-2 protein expression was performed using Western blot and immunocytochemistry. The results obtained using these two methods are consistent. Cadmium at all concentrations tested in this study did not alter significantly COX-2 protein expression (Fig. 3b and c). The images taken by fluorescence microscopy confirmed no effect of cadmium solution on COX-2 protein expression (Fig. 4).

### Prostaglandin E_2_ Production Is Unaltered by Cadmium Treatment in THP-1 Macrophages

**Fig. 5:**
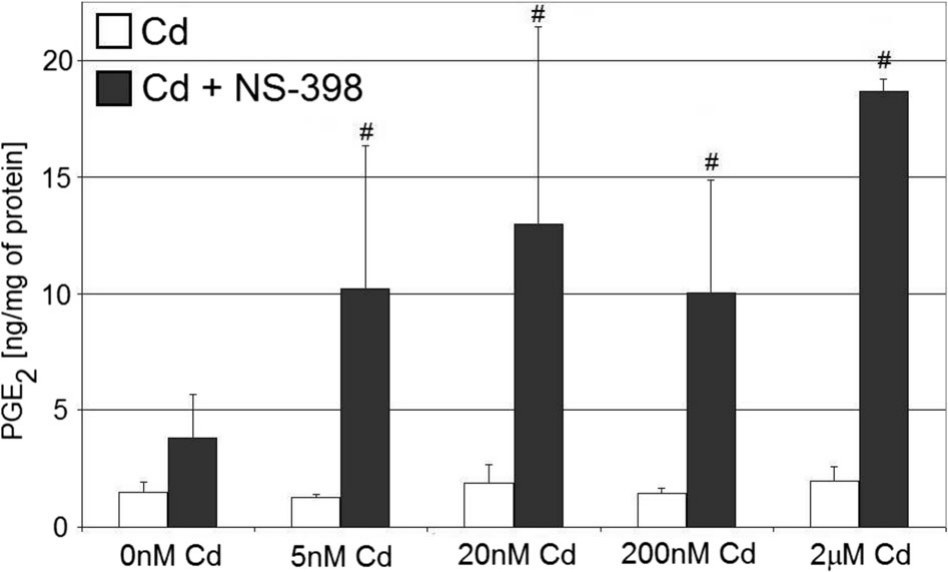
The effect of cadmium on quantity of PGE_2_ in culture supernatants of macrophages cultured with various cadmium solutions. Monocytes/macrophages were cultured with cadmium solutions for 48 h. After incubation, cells were harvested by scraping and PGE_2_ concentration was measured by ELISA method (*n* = 6). *Number sign*, statistically significant as compared with the experiment 0 nM Cd with NS-398 (Wilcoxon test)

Cadmium used in this study did not significantly affect PGE_2_ concentrations as compared to control (Fig. 5).

However, the treatment of THP-1 macrophages with both cadmium and NS-398 caused significant dose-dependent increase (262 to 488 %) in PGE_2_ concentration as compared to control (*p* = 0.027 for 5 nM; *p* = 0.027 for 20 nM; *p* = 0.043 for 200 nM; and *p* = 0.043 for 2 μM cadmium solutions).

### Cadmium at All Tested Concentrations Does Not Significantly Affect Thromboxane A_2_ Production in THP-1 Macrophages

**Fig. 6:**
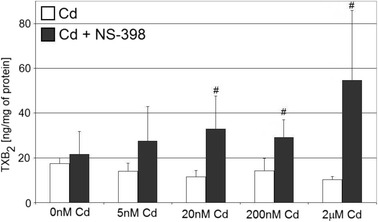
The effect of cadmium on quantity of TXB_2_ in culture supernatants of macrophages cultured with various cadmium solutions. Monocytes/macrophages were cultured with cadmium solutions for 48 h. After incubation, cells were harvested by scraping and TXB_2_ concentration was measured by ELISA method (*n* = 6). *Number sign*, statistically significant as compared with the experiment 0 nM Cd with NS-398 (Wilcoxon test)

Treatment of THP-1 macrophages at 200 and 2,000 nM of cadmium resulted in insignificant decrease (19 to 41 %) in TXB_2_ concentrations as compared to control (Fig. 6). However, co-incubation of macrophages with cadmium and selective COX-2 inhibitor, NS-398, resulted in the opposite effect, that is increase (28 to 153 %) in TXB_2_ concentration, with the highest tested cadmium concentration (2 μM Cd) causing marked increase (153 %) in TXB_2_ level, as compared to control (*p* = 0.043). Addition of NS-398 to cadmium treated macrophages’ cultures caused significant increase in TXB_2_ production for 20 nM (*p* = 0.05), 200 nM (*p* = 0.05), and 2 μM (*p* = 0.043) cadmium solutions.

## Discussion

The current study, to the best of our knowledge, is the first such study in which the effects of very low and low concentrations of cadmium (comparable to those occurring in the blood serum of general population or occupationally exposed workers) on inflammatory enzymes such as COX-1 and COX-2 and their products were analyzed using THP-1 macrophage experimental system.

### Cadmium and Cyclooxygenases mRNA Expression

We demonstrated that 48 h treatment of THP-1 macrophages with 2 μM cadmium significantly increased COX-1 mRNA expression. Similar results were reported by Miyahara et al. who showed that cadmium at 1 μM and above significantly increased the level of COX-1 mRNA in primary mouse osteoblastic cells [17]. The opposite results were reported by Figueiredo-Pereira et al.; the authors demonstrated that COX-1 gene expression was not upregulated by cadmium treatment (3–30 μM) in HT4 mouse neuronal cells [16].

The observed increased COX-1 mRNA expression due to the highest tested cadmium concentration may be explained by several mechanisms. COX-1 gene regulatory elements include three SP1 binding sites and AP-1 binding site [7]. Cadmium, in concentration range 0.5–20 μM, was found to induce the expression of c-fos and c-jun genes (genes that constitute AP-1 transcription factor) in different biological systems [30, 31]. We speculate that 48 h exposure of macrophages to 2 μM Cd might significantly activate AP-1 transcription factor, which in turn could significantly induce COX-1 gene promoter through enhanced binding AP-1 to DNA, resulting in increased COX-1 mRNA levels. Another possible mechanism responsible for increased COX-1 mRNA expression due to cadmium may be cadmium effects on secondary messengers, such as ROS or intracellular Ca^2+^ [10]. Although cadmium is not a Fenton metal, it causes generation of ROS; elevated levels of ROS might affect the redox-sensitive transcription factor AP-1, which could bind to appropriate COX-1 gene promoter regulatory element, and thus stimulate COX-1 mRNA expression [31]. Moreover, cadmium might activate protein kinases (such as protein kinase C) through the increased levels of intracellular Ca^2+^, resulting in enhanced phosphorylation of AP-1 transcription factor leading to transcriptional activation of COX-1 gene [10, 32].

Addition of NS-398 to macrophage cultures treated with cadmium resulted in significant increase in COX-1 mRNA expression (for 20 nM and 2 μM Cd). Selective COX-2 inhibitor NS-398 seems to enhance the stimulatory effect of cadmium on COX-1 mRNA levels.

Cadmium at concentrations 200 nM and 2 μM markedly upregulated COX-2 mRNA expression in THP-1 macrophages treated for 48 h. These results are in agreement with the results of the studies of Miyahara et al. [17], Figueiredo-Pereira et al. [16], Shin et al. [19], Seok et al. [18], and Park et al. [8]; however, it should be stressed that other experimental systems and usually higher cadmium concentrations were used by those authors. In contrast, two other studies showed the downregulation of COX-2 mRNA expression by cadmium [14, 15].

With respect to COX-2 gene transcriptional regulation, its promoter contains many *cis*-acting regulatory elements, of them only NF-κB site, CRE, NF-IL6 motifs, and E-box are known to be involved in the regulation of COX-2 gene expression [9, 33]. Transcription factors bind to these sites in a variety of combinations depending on cell type and also which regulatory pathway is activated [9]. Cadmium was found to activate NF-κB transcription factor [31]. We speculate that cadmium in higher tested concentrations (i.e., 200 nM and 2 μM) might cause marked upregulation of NF-κB, enhanced binding of NF-κB to COX-2 promoter, and COX-2 promoter activation leading to increased COX-2 mRNA expression. Similarly to COX-1 gene, also other mechanisms may be responsible for increased expression of COX-2 gene by cadmium, including effects on secondary messengers (ROS, intracellular Ca^2+^) and effects on signal transduction cascades involving kinases [10, 31]. For example, cadmium appeared to activate the following kinases: protein kinase C, mitogen-activated protein kinase (MAPK) family (ERK, JNK, p38), stress-activated protein kinase, casein kinase 2, calcium/calmodulin-dependent kinase II [31].

In our study, incubation of macrophages with cadmium and COX-2 selective inhibitor, NS-398, did not change significantly the COX-2 mRNA expression at most Cd concentrations tested; however, the lowest Cd concentration with NS-398 caused significant downregulation of COX-2 mRNA level. NS-398 appears to counteract the stimulatory effect of Cd on COX-2 mRNA levels. Our results seem to be in concert with the report of Callejas et al., who found that the interaction of NS-398 and LPS on COX-2 upregulation was not observed at the mRNA level [34]. In contrast to this, Blais et al. demonstrated that NS-398 enhanced the effects of LPS on transcriptional activation of key inflammatory molecules [35].

### Cadmium and Cyclooxygenase-1 and Cyclooxygenase-2 Protein Expression

The expression of COX-1 protein in THP-1 macrophages, as determined by Western blotting, decreased significantly following 48 h exposure to 20 and 200 nM Cd. Unfortunately, no studies have been found in the literature which dealt specifically with the effects of cadmium on COX-1 at protein level. Barrios-Rodiles et al. demonstrated that the level of COX-1 remained unaltered in PMA-differentiated THP-1 macrophages stimulated by other inflammatory stimulant which is LPS itself and pretreated with NS-398 [36]. Maybe the reason for the scarce of reports was the former opinion that COX-1 is a constitutive enzyme and does not change during inflammation [4]. However, this opinion appeared to be simplistic. Later on, there was suggestion that COX-1 contributes to inflammatory response [5], so the recent view is that both COX-1 and COX-2 enzymes show their activities under both physiological and pathological conditions, such as inflammation [5, 6].

As mentioned previously, cadmium at concentrations 20 and 200 nM exerted inhibitory effect on COX-1 protein level in THP-1 macrophages despite the absence of alterations in COX-1 mRNA level. The observed discrepancy between COX-1 mRNA and protein levels is not unique, since Gry et al. compared mRNA and protein profiles of 1,066 gene products in 23 human cell lines and found significant correlations only in one third of examined mRNA species and corresponding proteins [37]. This rather weak correlation between COX-1 mRNA and protein levels suggests the existence of some nonspecific effect of cadmium resulting in suppression of COX-1 mRNA translation. Probably, the possible mechanisms of COX-1 protein downregulation by cadmium might involve the following: changes in its mRNA stability [9], enhanced COX-1 degradation [7], interaction with COX-1 mRNA binding proteins leading to inhibition of COX-1 protein synthesis [38], effects on translation factors [39] (for example, CdCl_2_ caused significant decrease in protein level of translation initiation factor 4E, eIF4E). Similar regulatory mechanisms may also be responsible for the lack of marked alterations in COX-1 protein expression due at 2 μM cadmium concentration despite significant upregulation of COX-1 mRNA levels [9, 33, 40].

In our study, the expression of COX-2 protein in THP-1 macrophages was not significantly modulated by cadmium at all tested concentrations. This result is in agreement with the results obtained by Alvarez et al. who showed that cadmium (given to rats in concentration of 15 ppm in drinking water for 3 months) did not modify the expression of COX-2 in rat prostate [20]. Ahn et al. reported even suppression of COX-2 expression induced by LPS in RAW 264.7 macrophages by cadmium [14]. However, it is worth mentioning that most of available reports demonstrated significant increase in COX-2 protein expression due to cadmium exposure [16, 18, 21–25].

The observed absence of alterations in COX-2 protein levels despite significant upregulation of its mRNA following 48 h exposure to 200 nM and 2 μM Cd remains to be elucidated. However, one must remember that the regulation of COX-2 gene expression is very complex, and this heavy metal may modulate many regulatory mechanisms that operate at different levels [30, 38]. We may not exclude cadmium-mediated suppression of COX-2 protein translation, mechanisms of which might be similar to those described by us as potential modulators of COX-1 protein translation. We speculate that the possible effects of cadmium on COX-2 protein levels might involve the following: changes in COX-2 mRNA stability [33], the effect on RNA-binding proteins [9], the effect on miRNAs [7, 41], enhanced COX-2 degradation [7, 38], the effect on translation factors [7, 39].

### Cadmium and Prostaglandin E_2_ Synthesis by THP-1 Macrophages

Cadmium at the concentrations 5 nM–2 μM did not significantly modify the PGE_2_ levels in THP-1 macrophages medium following 48 h exposure. This result is in contrast to other study results which demonstrated stimulatory effect of cadmium (in general in concentrations of 1 μM and above) on PGE_2_ production by different cells like HT4 mouse neuronal cells [16], murine cerebrovascular endothelial cells [18], mouse osteoblasts [17, 26], neonatal mouse calvaria [27], and mouse peritoneal macrophages from cadmium-exposed mice (15 ppm Cd through drinking water for 2 months) [24].

In our study, co-incubation of macrophages with cadmium and selective COX-2 inhibitor NS-398 for 48 h resulted in significant dose-dependent increase in PGE_2_ concentration. This was an unexpected result, since previous reports showed inhibitory action of NS-398 on cadmium-stimulated PGE_2_ production in other cell lines [17, 27]. Maybe the increase in PGE_2_ levels following NS-398 treatment in cadmium-exposed macrophages was the consequence of paradoxical effect of NS-398 in our culture system and experimental conditions.

### Cadmium and Thromboxane B_2_ Synthesis by THP-1 Macrophages

Cadmium at concentrations and exposure duration tested in our study did not significantly modulate TXB_2_ production by THP-1 macrophages. This result is in concert with report of Eisenmann and Miller who analyzed the effects of cadmium on human placental production of TXB_2_: two 12 h exposures to cadmium (40 and 100 μM) caused no significant effect on TXB_2_ levels [28].

The treatment of THP-1 macrophages with both 2 μM cadmium and NS-398 for 48 h resulted in increase in TXB_2_ concentration as compared with control. This may also be the consequence of paradoxical effect of NS-398 in our culture system. As was suggested by Ziemann et al., may be the loss of anti-inflammatory efficacy of COX-2 selective inhibitor NS-398 at higher doses occurs through paradoxical activation of NF-κB and subsequent induction of NF-κB-dependent pro-inflammatory genes [42]. Blais et al. demonstrated that COX-2 inhibition increases inflammatory response in the brain during systemic immune stimuli [35]. Gilroy and colleagues showed in carrageenan-induced inflammation model in rats that NS-398 significantly exacerbated inflammation at 48 h [43].

The fact that significantly increased COX-1 and COX-2 mRNA expression at 2 μM Cd were not accompanied with increased protein and enzymatic activity levels remains to be resolved. Sovago and Varnagy demonstrated that cadmium(II) ions may form complexes with all natural amino acids and peptides [44]. Among the most effective metal binding amino acids are cysteine, methionine, aspartic acid, and histidine [44]. Twenty-four amino acid residues were found to line cyclooxygenase active site with only one difference between COX-1 and COX-2 [45]. Maybe the lack of alterations in COX-1 and COX-2 enzymatic activities is associated with cadmium interaction with some amino acid residues that constitute the active sites of these enzymes, leading to enzyme conformational changes affecting the catalytic efficiency of cyclooxygenases through decreased substrate binding.

## Conclusion

In conclusion, our study demonstrates that cadmium at the highest tested concentrations modulates COX-1 and COX-2 only at mRNA level in THP-1 macrophages; however, the lower tested cadmium concentrations appear to inhibit COX-1 protein expression. The stimulatory effect of cadmium on COXs at mRNA level is not reflected at protein and enzymatic activity levels, suggesting the existence of some posttranscriptional, translational, and posttranslational events that result in silencing of those genes’ expression.

Despite the minimal effect of cadmium on COXs found in this study, we may not exclude the possible cumulative effect of its action in such low concentrations, which will be the topic of our future research.
